# Influence of the Structure of Hydrothermal-Synthesized TiO_2_ Nanowires Formed by Annealing on the Photocatalytic Reduction of CO_2_ in H_2_O Vapor

**DOI:** 10.3390/nano14161370

**Published:** 2024-08-21

**Authors:** Andrey M. Tarasov, Larisa I. Sorokina, Daria A. Dronova, Olga Volovlikova, Alexey Yu. Trifonov, Sergey S. Itskov, Aleksey V. Tregubov, Elena N. Shabaeva, Ekaterina S. Zhurina, Sergey V. Dubkov, Dmitry V. Kozlov, Dmitry Gromov

**Affiliations:** 1 Institute of Advanced Materials and Technologies, National Research University of Electronic Technology MIET, Bld. 1, Shokin Square, Zelenograd, 124498 Moscow, Russiatrif123456@yandex.ru (A.Y.T.);; 2 National Research Centre “Kurchatov Institute”, 1 Kurchatov Square, 123182 Moscow, Russia; 3 S.P. Kapitsa Scientific Technological Research Institute, Ulyanovsk State University, 42 Leo Tolstoy Street, 432017 Ulyanovsk, Russia; 4 Institute for Bionic Technologies and Engineering, I.M. Sechenov First Moscow State Medical University, Bolshaya Pirogovskaya 2, 119435 Moscow, Russia

**Keywords:** hydrothermal synthesis, TiO_2_, photocatalysis, CO_2_ photoreduction

## Abstract

The present study investigates the photocatalytic properties of hydrothermally synthesized TiO_2_ nanowires (NWs) for CO_2_ reduction in H_2_O vapor. It has been demonstrated that TiO_2_ NWs, thermally treated at 500–700 °C, demonstrate an almost tenfold higher yield of products compared to the known commercial powder TiO_2_ P25. It has been found that the best material is a combination of anatase, TiO_2_-B and rutile. The product yield increases with increasing heat treatment temperature of TiO_2_ NWs. This is associated with an increase in the degree of crystallinity of the material. It is shown that the best product yield of the CO_2_ reduction in H_2_O vapor is achieved when the TiO_2_ NW photocatalyst is heated to 100 °C.

## 1. Introduction

TiO_2_ is a semiconductor material that is widely used in photocatalysis. It is distinguished by its chemical stability, superhydrophilicity, durability, low cost, lack of toxicity to humans and transparency in the visible range of the spectrum. The photocatalytic properties of TiO_2_ are explained by the formation of charge carriers when exposed to photons with a wavelength of less than 400 nm, which is due to the optical band gap of about 3.2 eV [1]. TiO_2_ finds its application in self-cleaning surfaces [2,3], it is used to purify water [4,5] and air [6,7], as well as in the decomposition of water into oxygen and hydrogen [8,9]. The strong oxidizing properties of TiO_2_ make it possible to use it for the decomposition of many organic compounds [10]. In addition to the ability to oxidize a number of substances on its surface, TiO_2_ is capable of reducing CO_2_ to a number of hydrocarbons [11,12].

The morphology of TiO_2_ nanoparticles can have a great influence on the properties of the resulting structures. Among the many variations of morphology are nanospheres [13,14], nanowires [15,16], nanotubes [17,18] and nanosheets [19,20]. It is known that the following parameters are important for a nanostructure used in photocatalysis: the shape of the nanostructure [21,22], the specific surface area [23,24], the crystalline phase [25,26] and the lifetime of charge carriers [27,28].

One of the most interesting structures for use in photocatalysis are nanowires, which combine the advantages of zero- and two-dimensional objects [29]. Compared to zero-dimensional objects, nanowires have a high aspect ratio, which increases the efficiency of absorption of incident radiation by a single crystal. In addition, the long length and high crystallinity reduce the probability of charge carrier recombination, and the geometry promotes diffusion-free transport of charge carriers along the axial direction of the crystal [30,31]. As a result, it has been repeatedly shown that one-dimensional TiO_2_ exhibits higher photocatalytic activity than TiO_2_ powders [32,33]. However, there is also evidence indicating the opposite results, where nanoparticles demonstrate higher photocatalytic activity [33]. These conflicting results highlight the complexity and diversity of factors affecting photocatalytic activity and the need for further research to gain a deeper understanding of these processes.

In this work, TiO_2_ nanowires were used for artificial photosynthesis—the reduction of CO_2_ when interacting with water under the influence of light. It is demonstrated that TiO_2_ nanowires formed by the hydrothermal method are more effective for this purpose than commercial TiO_2_ P25. However, this efficiency depends on the crystal structure of the nanowires, determined by the high-temperature post-processing conditions.

## 2. Materials and Methods

TiO_2_ nanowires were obtained by hydrothermal synthesis. A total of 50 mL of a 10 M aqueous NaOH solution was placed in a stainless autoclave with a 100 mL Teflon container. Then, 0.6 g of commercial TiO_2_ powder P25 (Evonik, Hanau, Germany) was added to the prepared solution and stirred using a magnetic stirrer for 30 min. After stirring, the autoclave was heated in a muffle furnace to 250 °C and kept there for 9 h. The autoclave cooled to room temperature inside the muffle furnace. The resulting nanowires were placed in 400 mL of deionized water and stirred with heating to 60 °C. Then, 50 mL HCl was added and stirred for 20 min. The nanowires were washed repeatedly in deionized water using vacuum filtration until a neutral pH was reached. Drying of the nanowires was carried out in air at a temperature of 100 °C.

Temperature post-processing of the samples was carried out by annealing in air at temperatures of 350, 500, 700 and 900 °C. Annealing was carried out for 4 h.

Adsorption–desorption isotherms were obtained using the instrument Quantachrome Nova 3200e (software NovaWin V.11.03 1994—2013 Quantachrome Instr., Boynton Beach, FL, USA). Specific surface area was calculated using the BET (Brunauer–Emmett–Teller) method in the relative pressure range from 0.05 to 0.25. The samples were preliminarily degassed at 300 °C for 3 h.

**Figure 1:**
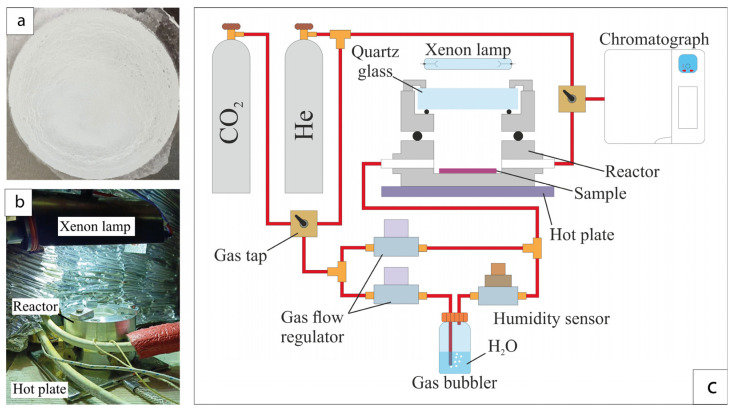
A photograph of nanowire samples on a titanium substrate (**a**), the reactor appearance (**b**) and a diagram of the stand for measuring the photocatalytic activity of the samples (**c**).

To perform photocatalytic studies, a layer of TiO_2_ nanowires with an area of 7 cm^2^ and a specific mass of about 1.5 mg/cm^2^ was deposited onto a titanium foil substrate using the drop method (Figure 1a). The drop method of forming layers of TiO_2_ nanowires involved applying an aqueous suspension onto titanium foil. For this purpose, a suspension was prepared consisting of 1.5 mL of deionized water and about 15 mg of TiO_2_ nanowires. The suspension was treated in an ultrasonic bath for 30 s. After preparation, the suspension was placed in a medical syringe and applied to the surface of the prepared titanium foil. To prevent the suspension from spreading, a silicone ring with an internal diameter of about 30 mm was used. Then, the suspension was dried on a heating table at a temperature of about 85 °C. Titanium foil was preliminarily degreased by ultrasonic treatment in a mixture of acetone/isopropyl alcohol (1:1) for 30 min. After degreasing, the foil was etched in a solution HF:HNO_3_:H_2_O (30:20:150).

The photocatalytic activity of the samples was studied in a thermally stabilized flow reactor with a volume of 25 cm^2^ with a quartz window and two gas inlets (Figure 1b). The samples were irradiated using two 35 W xenon lamps. The measurements were carried out at temperatures of 40, 70, 100 and 125 °C. The incoming reaction gas mixture consisted of 40 vol.% CO_2_ and 60 vol.% H_2_O and was supplied at a gas flow rate of 3 mL/min. Helium was used as the carrier gas. The amount of water entering the reactor was estimated using a gas humidity sensor DV2TSM V. Humidity was changed by passing a CO_2_ flow through a bubbler (Figure 1c). The gas flow regulator controlled the flow of CO_2_. The Crystal 5000 gas chromatograph analyzed the products of the photocatalytic reaction. The chromatograph was equipped with an Agilent HP PLOT Q capillary column and a plasma ionization detector. The reaction products were identified by the exit time. The product concentration was estimated from the peak area. Before studying the sample, the reactor was heated to 60 °C and purged for one hour. After installing the sample into the reactor, the product yield was recorded in the dark three times in a row. The resulting base values were subtracted when further calculating the product yield.

The morphology of nanowires was studied using a Helios G4 CX (FEI company, Hillsboro, OR, USA) scanning electron microscope (SEM). The measurements were carried out at an accelerating voltage of about 5 kV and a current of 21 pA.

X-ray diffraction (XRD) analysis was carried out using the diffractometer Rigaku MiniFlex 600 (Rigaku Corporation, Tokyo, Japan) with radiation source Cu Kα (λ = 1.5418 Å). The Bragg angle 2θ ranged from 10° to 60°.

Transmission electron microscopy (TEM) studies were carried out using an Tecnai G2 microscope (FEI company, Hillsboro, OR, USA) with an accelerating voltage of 200 kV with an EDAX energy dispersive X-ray analysis attachment.

Raman spectroscopy of the samples was performed using a high-precision Confotec MR200 microscope (SOL instruments, Minsk, Belarus). The sample was targeted using a near-focus MPlanFL N 40× objective (Olympus, Tokyo, Japan). A laser with a wavelength of 532 nm and a power of 440 μW was used for the measurements. The diameter of the focused laser beam was 1.5 μm. A diffraction grating of 600 lines/mm and a registration pinhole of 100 μm were used for the measurements. The measurements were performed in the mapping mode in the area of 10 × 10 μm, the number of measurement points was 5 × 5 (25 in total), the step between the points was 2 μm, and the spectrum acquisition time at each point was 1 s. After the measurement was completed, the aggregated spectrum from all measured points was saved.

## 3. Results

### 3.1. SEM Study of Morphology of Experimental Samples

**Figure 2:**
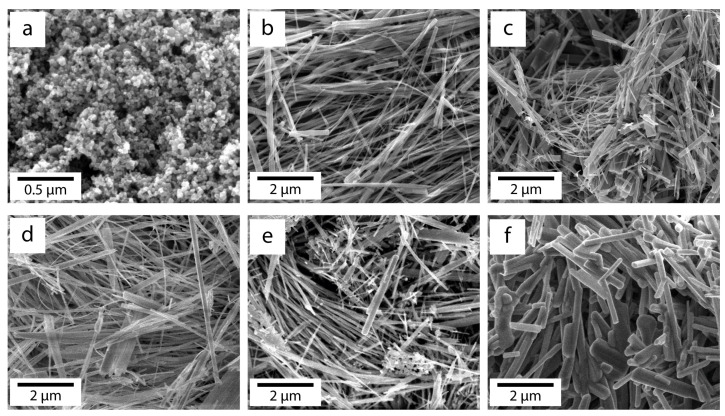
SEM photo of morphology of TiO_2_. (**a**) Commercial P25, TiO_2_ nanowires; (**b**) original sample; (**c**) after annealing at 350 °C; (**d**) after annealing at 500 °C; (**e**) after annealing at 700 °C; and (**f**) after annealing at 900 °C.

SEM images of commercial TiO_2_ P25 and nanowires obtained by hydrothermal reaction and subjected to heat treatment at different temperatures are shown in Figure 2. Commercial powder P25 (Figure 2a) consists of spherical particles with an average size of about 25 nm. The synthesized nanowires have lengths ranging from 6 to 10 μm and thicknesses from 30 to 300 nm. Heat treatment at temperatures ranging from 350 to 700 °C leads to insignificant changes in morphology. The most noticeable modification is observed in nanowires annealed at 900 °C for 4 h. As can be seen in Figure 2e, in this case, the diameter of the nanowires increases, and the ends of the nanowires take a more rounded shape.

### 3.2. XRD Studies of Phase Composition of Experimental Samples

**Figure 3:**
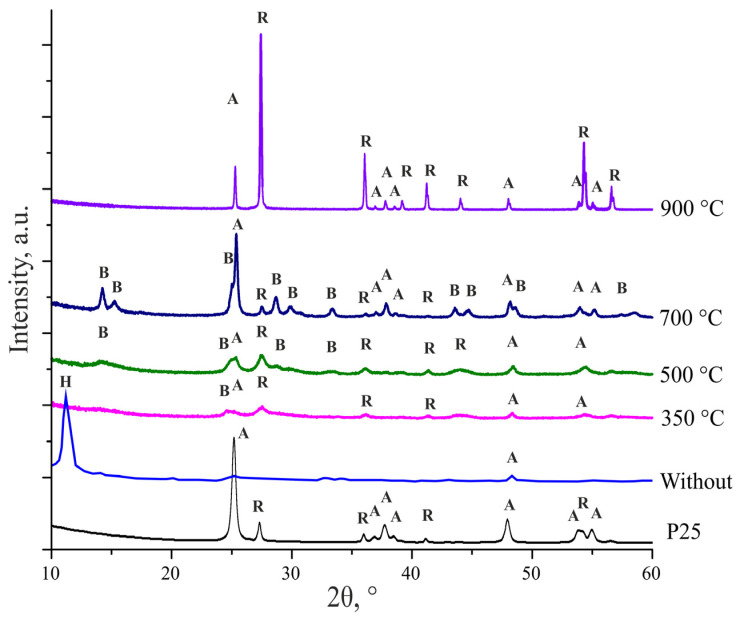
XRD patterns of TiO_2_ nanowire samples. Phase designation: H—hydrogen titanate, A—anatase, R—rutile, and B—bronze (TiO_2_-B).

Figure 3 shows X-ray diffraction patterns of TiO_2_ nanowires, initial and heat-treated at different temperatures. After synthesis and washing in acid, nanowires have low crystallinity. The X-ray diffraction pattern of this sample contains almost one pronounced peak at 12°, which is usually identified as titanate or H_2_TiO_3_ [34]. After the initial nanowires were subjected to heat treatment at 350 °C for 4 h, the appearance of weakly intense and broadened peaks of different phases of titanium dioxide is observed: TiO_2_-B (“bronze”), anatase and rutile. Heat treatment in the range of 350–700 °C does not cause a change in the phase composition but leads to the fact that the peaks of the TiO_2_-B, anatase and rutile phases become more pronounced, and their intensity increases. A change in the phase composition is observed after heat treatment at 900 °C. Reflections of the TiO_2_-B phase completely disappear. The rutile phase becomes predominant. However, weak peaks characteristic of anatase still remain.

### 3.3. TEM Study of Structure and Phase Composition of Experimental Samples

**Figure 4:**
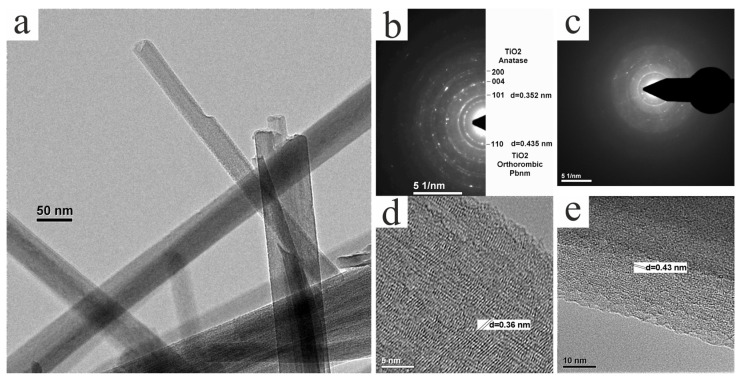
Results of TEM studies of initially synthesized nanowires: (**a**) TEM image of nanowires; (**b**) selected area diffraction pattern (SADP); (**c**) SADP after 10 min of exposure to high-intensity electron beam; and (**d**,**e**) high-resolution TEM (HRTEM) images.

**Figure 5:**
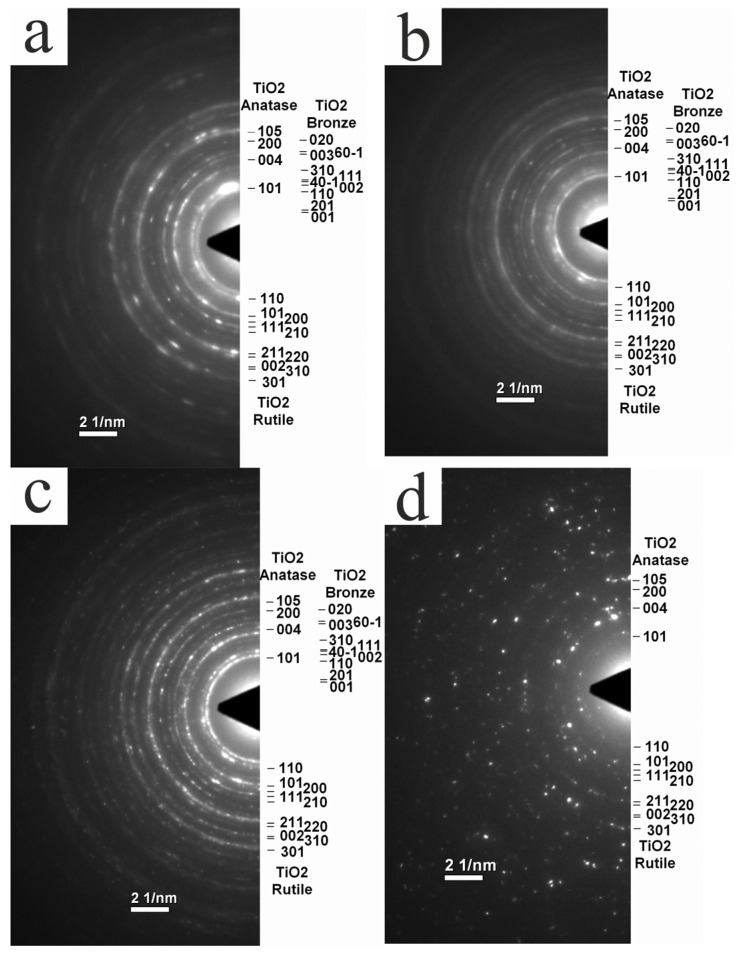
Selected area diffraction patterns (SADPs) of nanowire samples after temperature treatment: (**a**) 350 °C; (**b**) 500 °C; (**c**) 700 °C; and (**d**) 900 °C.

**Figure 6:**
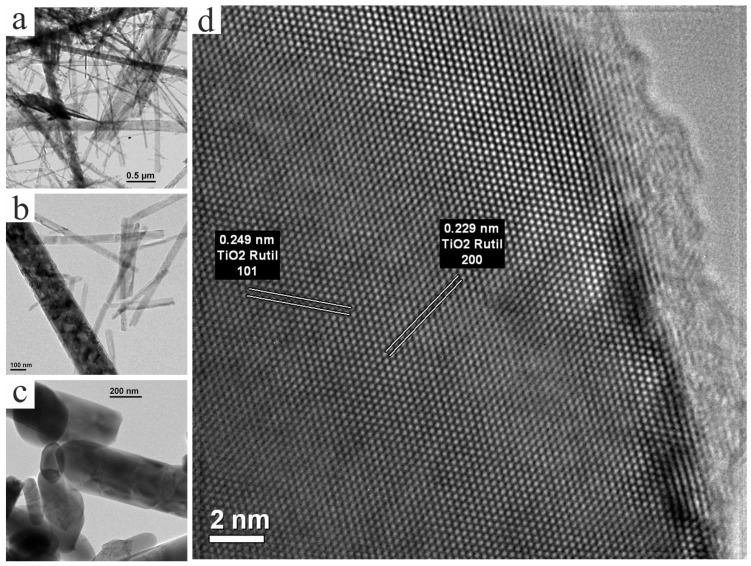
TEM images of samples after temperature treatment: (**a**) 700 °C at 7k× magnification; (**b**) 700 °C at 20k× magnification; (**c**) 900 °C at 20k× magnification; and (**d**) 900 °C at 1M× magnification.

**Figure 7:**
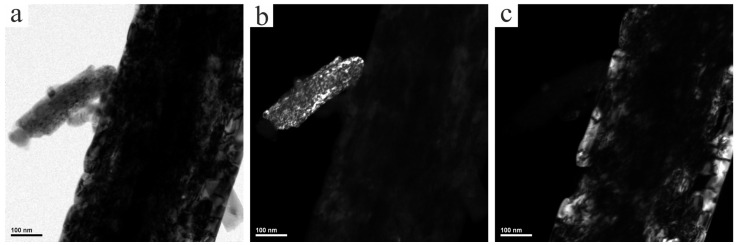
TEM images of sample fragments annealed at 700 °C in bright-field mode (**a**) and dark-field mode in electrons scattered by (101) planes of anatase (**b**) and (110) planes of rutile (**c**).

Figure 4, Figure 5, Figure 6 and Figure 7 show the results of the studies of the initial and annealed samples using transmission electron microscopy.

Elemental analysis of the initial synthesized nanowires by the EDX method showed that the spectrum contains only peaks corresponding to titanium and oxygen. In the diffraction patterns of this sample (Figure 4c,d), reflections corresponding to two interplanar spaces, ~0.35 nm and ~0.43 nm, are most pronounced. The first of them corresponds to the most intense planes (101) of the tetragonal structure of anatase: d = 0.352 nm. Anatase crystallites are also visible in the high-resolution images in Figure 4d,e.

Among the numerous modifications of titanium oxide, the interplanar spacing d = 0.435 nm is typical only for the (110) planes of the orthorhombic phase TiO_2_ with the symmetry group Pbnm [35]. All other diffraction reflections in this structure have very low intensity; therefore, in the diffraction patterns in Figure 4, only planes (110) are indexed. The high-resolution image in Figure 4e shows that these planes are oriented along nanowires.

The diffraction pattern in Figure 4b is slightly different from the XRD data. In particular, there are no reflections characteristic of hydrogen titanate. This may be caused by the low stability of titanate and may also be associated with both a long period of exposure of the samples to air and exposure to a high-energy electron beam of the microscope. It should be noted that after a long stay of this sample under the electron beam, reflections with high indices almost disappear in the diffraction pattern (Figure 4c), and diffuse rings corresponding to the amorphous phase become more intense.

Selected area diffraction patterns (SADPs) shown in Figure 5 demonstrate the evolution of the crystal structure of the samples after annealing. In samples annealed at 350, 500 and 700 °C, the diffraction pattern consists of rings corresponding to the crystal lattices of anatase, rutile and TiO_2_-B, as well as diffuse rings of the amorphous phase. The rings corresponding to TiO_2_-B appear rather weakly, and the contribution of the amorphous phase weakens as the temperature increases. The diffraction pattern in a sample heated to 900 °C has a different character: instead of rings, it has a system of individual diffraction spots, which generally correspond to the crystal lattice of rutile. This result is quite consistent with TEM and HRTEM images of this sample (Figure 6c,d). Its nanostructures are rutile single crystals elongated along the [001] crystalline axis.

As for the nanostructures of the samples annealed at 350, 500 and 700 °C, they are similar to each other. Their typical appearance is shown in Figure 6a,b. It can be seen that the material under study can be considered as a mixture of thin fibers and thick nanowires. Studying these objects in a dark-field mode (Figure 7) allows us to conclude that thin fibers in their mass have an anatase crystalline structure, and large nanowires have a rutile structure.

### 3.4. Raman Studies of the Phase Composition of Experimental Samples

**Figure 8:**
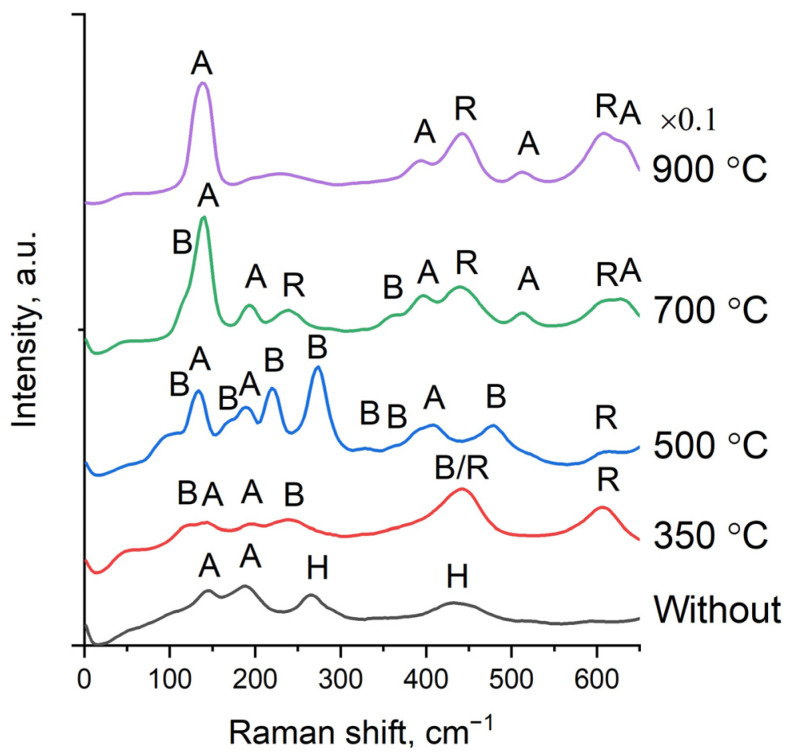
Raman spectra of TiO_2_ nanowire samples. Phase designation: H—hydrogen titanate, A—anatase, R—rutile, and B—bronze (TiO_2_-B).

Figure 8 shows the Raman spectra of TiO_2_ nanowires, both original and thermally treated at different temperatures. The sample that has not been subjected to heat treatment is characterized by low-intensity peaks. The observed peaks are identified as hydrogen titanate [36] and anatase [37]. After heat treatment, the hydrogen titanate peaks disappear. At the same time, peaks characteristic of rutile [37], anatase and bronze [38] appear. The highest intensity of the peaks is observed after annealing at 900 °C, which is due to the highest crystallinity of the nanowires. At this temperature, only anatase and rutile peaks are present. The Raman spectroscopy results generally confirm the results obtained using XRD and TEM.

### 3.5. BET Studies of the Specific Surface Area of Experimental Samples

**Figure 9:**
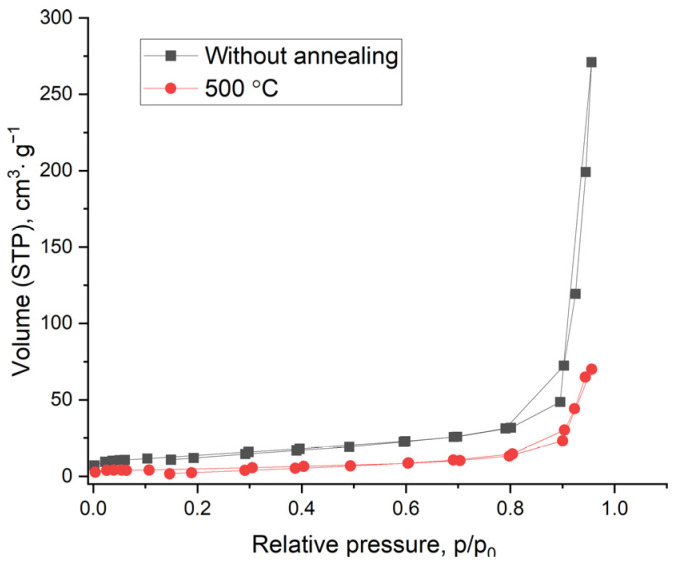
Nitrogen adsorption–desorption isotherms at 77 K of TiO_2_ nanowires without annealing and after annealing at 500 °C.

To analyze the specific surface area of TiO_2_ nanowires, the samples were studied by the capillary nitrogen condensation method. Figure 9 shows nitrogen adsorption–desorption isotherms at 77 K for the initial sample of TiO_2_ nanowires and after annealing at 500 °C. Capillary condensation hysteresis on isotherms indicates the presence of mesopores in TiO_2_ nanowires.

Table 1 presents the values of the specific surface area *S* calculated based on the analysis of isotherms using the BET method.

The commercial TiO_2_ P25 shows the highest specific surface area. At the same time, the specific surface area of TiO_2_ nanowires is lower and decreases with increasing annealing temperature. Synthesized nanowires that have not undergone heat treatment have the largest surface area. After heat treatment at a temperature of 900 °C, the specific surface area decreases by 15 times.

### 3.6. Optical Properties

**Figure 10:**
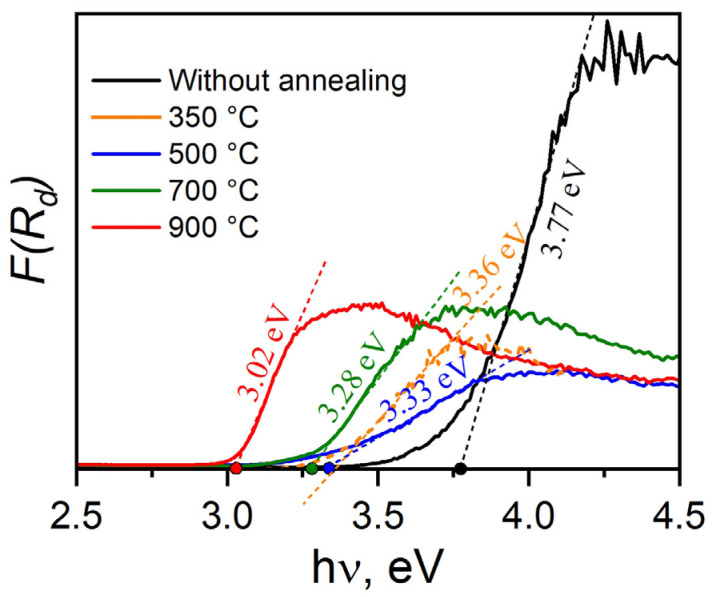
Results of Kubelka–Munk theory calculations for TiO_2_ nanowires.

The optical properties of TiO_2_ nanowire samples were studied by measuring the spectral dependences of diffuse reflectance in the UV and visible regions of the spectrum. From the diffuse reflectance data, the spectral absorption dependences of the samples were obtained using the Kubelka–Munk function [39]:

(1) $$F\left(R_{d}\right)=\frac{{\left(1-R_{d}\right)}^{2}}{2R_{d}}$$

where *R_d_* is the diffuse reflection coefficient, and *F*(*R_d_*) is a value proportional to the absorption coefficient. The optical band gap of all samples was estimated by determining the point of intersection of the tangent drawn to the linear portion of the dependence of *F*(*R_d_*) on energy with the abscissa axis as shown in Figure 10.

The original nanowires have the largest optical band gap. A similar value was obtained for hydrogen titanate [40]. For annealed samples, a noticeable decrease in the optical band gap is observed. However, as can be seen in Figure 9, in the heat treatment range of 350–700 °C, it does not change so noticeably within the range of 3.36–3.28 eV. Obviously, this is due to the appearance of a mixture of crystalline phases of TiO_2_-B, rutile and anatase. However, the optical band gap still remains larger than that of any of these emerging crystalline phases, having an *E_g_* of 2.8, 3.0 and 3.2 eV, respectively. [38,39,40,41,42,43]. Only the sample annealed at 900 °C showed an optical band gap of 3.02 eV, which is close to pure rutile.

### 3.7. Analysis of Structural Characterization of TiO_2_ Nanowire Samples

A comprehensive comparison of research results using various methods allows us to obtain a completely clear picture of the structure of the synthesized TiO_2_ nanowires and their changes as a result of heat treatment at different temperatures (Table 2).

Despite some discrepancies in the XRD and TEM data for the initially synthesized nanowires, it can be concluded that as a material they are weakly crystalline, highly defective and unstable. This material has the largest optical band gap, but at the same time, the largest specific surface area compared to heat-treated samples. One can hardly expect stable properties and reliable operation from such a material.

Thermal treatment of the material in the range of 350–700 °C leads to the formation of multiphase TiO_2_ nanowires containing anatase, bronze and rutile phases. At the same time, we believe that after such treatment, the amorphous phase remains in some quantity. This is indicated by the optical band gap of these samples, which turns out to be larger than that of anatase, rutile or bronze (Table 2). An increase in the material processing temperature in the specified range generally does not change the phase composition of TiO_2_ NWs but changes the quantitative ratio of the phases and increases the crystallinity of the material. This is evidenced by an increase in the intensity of the XRD peaks and the sharpness of the TEM diffraction pattern (the transformation of continuous diffuse diffraction rings into sharp rings consisting of a set of point reflections)

In this regard, from the X-ray diffraction patterns of the samples (Figure 3), the average crystallite size of the material was estimated using the Scherrer formula:

(2) $$D_{p}=\frac{0.94\cdot \lambda }{\beta \cdot \cos\theta }$$

where *D_p_*—average crystallite size, *β*—line broadening in radians, *θ*—Bragg angle, and *λ*—X-ray wavelength. The calculation results are presented in Table 2. It can be seen that the average crystallite size increases with increasing temperature of the heat treatment of the material.

Thus, increasing the heat treatment temperature in the range of 350–700 °C positively improves the crystal structure of the material, but in a negative way reduces its specific surface area (Table 2).

Analysis of the results of the sample annealed at atemperature of 900 °C shows that it is highly crystalline TiO_2_ NWs, in which the rutile phase predominates with some residual anatase content. However, at the same time, this material has the smallest specific surface area.

### 3.8. Photocatalytic Studies

**Figure 11:**
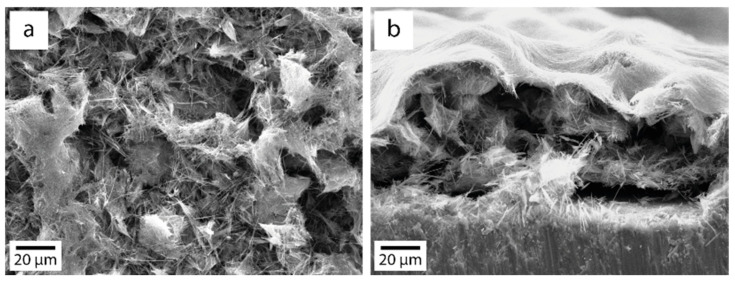
SEM images of a TiO_2_ NW-500 layer formed by the drop method: top (**a**) and side (**b**) views.

We used the obtained TiO_2_ NWs as a photocatalyst for the photocatalytic reduction of CO_2_ in H_2_O vapor. A series of TiO_2_ NW samples, thermally treated in the considered temperature range of 350–900 °C, were prepared by the drop method to study photocatalytic activity. The SEM image of the deposited layer of TiO_2_ NW-500 on titanium foil is shown in Figure 11. The figure shows that the layer has a pronounced, developed relief containing many mesopores.

**Figure 12:**
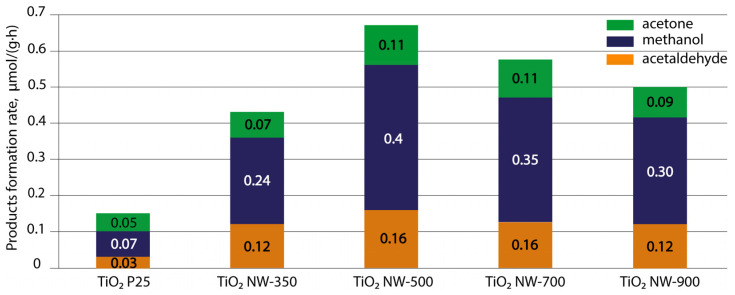
Average yield of CO_2_ photoreduction reaction products for layers of TiO_2_ NWs annealed at different temperatures and commercial TiO_2_ P25.

Figure 12 shows the change in the yield of CO_2_ photoreduction reaction products in the presence of water vapor depending on the mode of formation of the TiO_2_ NW photocatalyst materials. For comparison, the figure also shows the results obtained using the commercial TiO_2_ P25 powder photocatalyst.

It was found that the main products of CO_2_ photoreduction on the surface of TiO_2_ NWs and TiO_2_ P25 are methanol, acetone and acetaldehyde. In all cases, the predominant reaction product is methanol. As can be seen in Figure 11, the first thing that attracts attention is that the yield of CO_2_ photoreduction products when using TiO_2_ NWs as a photocatalyst is noticeably higher than when using commercial TiO_2_ P25, despite the higher specific surface area of the latter.

The second thing, as you can see in Figure 12, is that with increasing processing temperature, the yield of products first increased, reaching the highest value of 0.67 µmol/(g·h) for the TiO_2_ NW-500 sample, and then, with an increase in temperature to 900 °C (TiO_2_ NW-900), it decreased to 0.5 µmol/(g·h).

In the photocatalytic reduction of CO_2_ on TiO_2_, products are formed through the direct generation of VB-CB electron–hole pairs under the influence of UV light. In particular, methanol is formed under the influence of UV light according to the following scheme [44]:

(3) $$H_{2}O+h^{+}\to H^{+}+{OH}^{\cdot }$$

(4) $${CO}_{2}+{6H}^{+}+{6e}^{-}\to {CH}_{3}OH+H_{2}O$$

We attribute the higher yield of products on TiO_2_ NWs to two factors. Firstly, it is believed that one-dimensional TiO_2_ structures have higher charge carrier mobility and more efficient charge carrier transport along the axial direction. This fact was confirmed by DFT modeling for TiO_2_ nanobelts [45] and nanowires [31]. It was also demonstrated that TiO_2_ nanofibers generated three times higher photocurrent compared to nanoparticles and also exhibited enhanced photocatalytic activity for hydrogen production [32].

Secondly, the yield of photocatalytic process products is influenced by the heterostructural state of the material, when several phases with different band gap widths are in contact, which promotes the separation and accumulation of charge carriers, increases their lifetime and reduces the probability of recombination. It is known that the combination of different phases of titanium dioxide demonstrates significantly improved photocatalytic properties compared to single-phase titanium dioxide [46,47]. In our case, the best photocatalytic activity was demonstrated by the NW-500 TiO_2_ sample, which is a combination of anatase, rutile, bronze and amorphous phases. Some studies show an increase in photocatalytic activity in the presence of the bronze phase compared to the rutile/anatase combination [38]. Besides that, this kind of temperature dependence in Figure 12 can also be associated with the influence of two differently directed factors: an increase in crystallinity, which decreases the charge carrier recombination probability, on the one hand, and a decrease in the specific surface area on the other (Table 2). Thus, there is a certain optimum corresponding to a high degree of crystallinity, a large surface area and the presence of a heterophase transition. We believe that due to the described set of properties, the TiO_2_ NW-500 sample demonstrates the highest performance in the photocatalytic reduction of CO_2_.

**Figure 13:**
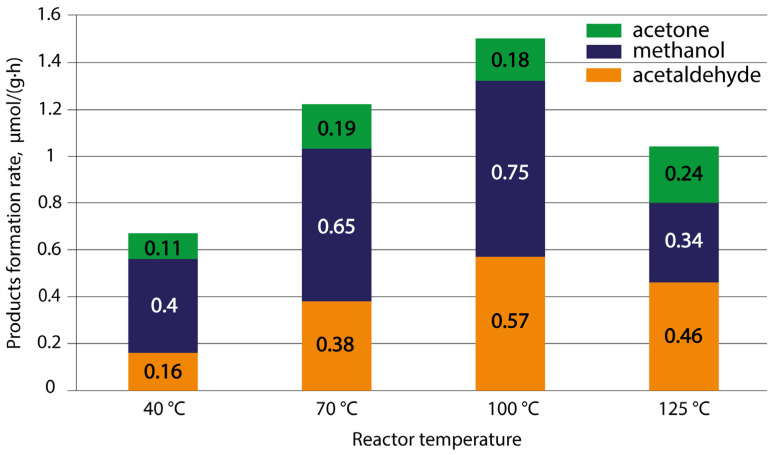
Average product yield in µmol/(g·h) for TiO_2_ NW-500.

Since the sample of TiO_2_ NW-500 proved to be the best photocatalyst, it was used to study the effect of the sample temperature in the reactor on the process of CO_2_ reduction in water vapor. The results of this study are shown in Figure 13.

Figure 13 shows that as the reactor temperature increases to 100 °C, photocatalytic activity increases, and with a further increase in temperature, the activity decreases. With an increase in temperature from 40 °C to 100 °C, the total yield of reaction products increased by 2.5 times. This trend of changes in photocatalytic activity with changes in temperature can be associated with a shift in the dynamic equilibrium between the adsorption and desorption of CO_2_ molecules and reaction products. Elevated temperature facilitates the desorption of products, providing a pathway for CO_2_ to adsorb onto vacant sites, resulting in increased reaction rates [48,49,50]. However, increasing the temperature to 125 °C already leads to a decrease in product yield. A similar character of the temperature dependence was found earlier in the study of RuNi/TiO_2_ for CO-tolerant toluene hydrogenation [51], in the investigation of the performances of Pd/TiO_2_ catalysts in the steam reduction of CO_2_ and TiO_2_ [52], and in the research of Pd/TiO_2_ photocatalysts using the example of methylene blue decomposition in water [53]. Taking into account these observations, we believe that this may be due to an unfavorable ratio of adsorption of precursors under conditions of simultaneous exposure of the surface to radiation, which leads to their insufficiency for the reaction. Thus, for TiO_2_ NWs synthesized and thermally treated at 500 °C, the highest catalytic activity is observed at a reactor temperature of 100 °C.

## 4. Conclusions

The present study shows that the hydrothermally synthesized TiO_2_ nanowire photocatalyst is much more effective for CO_2_ reduction in H_2_O vapor than the known powder commercial photocatalyst P25 (Evonik). This is determined by a combination of one-dimensional TiO_2_ structures, phase composition, crystallinity and the specific surface area of the material, which are controlled by thermal post-treatment of the initially synthesized TiO_2_ nanowires. In our case, the best result of the photocatalytic reduction of CO_2_ in H_2_O vapor is achieved at 500 °C annealing due to the storage of relatively high specific surface areas, the presence of the TiO_2_-B phase and high enough crystallinity. In addition, the photoreduction process itself of CO_2_ in H_2_O vapor should be optimized by both the irradiation and the surface temperature. Obviously, this combination of irradiation and temperature determines the conditions for the separation of pairs of charge carriers generated in TiO_2_ NWs under the irradiation, their lifetime and the conditions for the adsorption–desorption of reagents and products. In our study, using xenon lamp irradiation, the highest photocatalytic activity of TiO_2_ NWs is observed at the reactor temperature of 100 °C. Thus, expanding the understanding of the influence of the structural features of a material on its photocatalytic properties is useful for the development of green technologies, such as artificial photosynthesis.

## Figures and Tables

**Table 1:** Values of specific surface area of TiO_2_ nanowires at different annealing temperatures.

Annealing Mode	P25	No Annealing	350 °C	500 °C	700 °C	900 °C
*S*, m^2^/g	57.0	49.0	39.0	17.3	12.3	3.2

**Table 2:** Summary of research results.

Annealing	Sample Designation	Surface Area, m^2^/g	Optical Band Gap, eV	Crystallite Size, nm	Phases
No annealing	As-synthesized TiO_2_ NW	49.0	3.77	~12	Titanate, amorphous, anatase
350 °C	TiO_2_ NW-350	39.0	3.36	~13	Amorphous, anatase, bronze, rutile
500 °C	TiO_2_ NW-500	17.3	3.33	~16	Amorphous, anatase, bronze, rutile
700 °C	TiO_2_ NW-700	12.3	3.28	~26	Amorphous, anatase, bronze, rutile
900 °C	TiO_2_ NW-900	3.2	3.02	~63	Anatase, rutile

## Data Availability

The data presented in this study are available upon request from the corresponding author.
